# Deprescribing off label antipsychotic medications in a general adult psychiatry service: A risk management Perspective

**DOI:** 10.1192/j.eurpsy.2025.1596

**Published:** 2025-08-26

**Authors:** J. Khan

**Affiliations:** 1 Psychiatry, Connolly Hospital Blanchardstown, Dublin, Dublin, Ireland

## Abstract

**Introduction:**

Antipsychotic medications, initially designed for severe mental illnesses like schizophrenia and bipolar disorder, have witnessed a significant rise in off-label prescribing for a diverse range of conditions and at times these medications remain unchanged over long periods of time and poses a potential risk due to their side effect profile. There are different types of risks associated and depends on the dose of medication, age and comorbid physical health conditions of the patient.

**Objectives:**

To evaluate the outcomes of tapering and discontinuing off-label low-dose antipsychotic medications in patients without psychiatric disorders, focusing on the effectiveness of the deprescribing process and any associated withdrawal symptoms or adverse effects.

**Methods:**

The authors decided to review their caseload and identify patients who are prescribed off label use of antipsychotic medication for example for sleep issues, Axis 2 mental illness as per DSM-4 classification and on doses where there was no indication of antipsychotic medication. It was planned to have detailed discussion with patients about the impact of having off label antipsychotic prescription and agreed to slowly tapper off and discontinue this medication. There were 18 patients identified with off label use of antipsychotic medication. In this quasi-experimental study design, we selected eighteen patients by convenience sampling method from the caseload who were identified to be on off label prescription of antipsychotic medications. These patients were explained to the purpose of the study and the course of intervention, and all agreed to participate in the intervention.

**Results:**

Out of 40 patients prescribed off-label antipsychotic medications, 18 were identified for review, all had been receiving mental health services for over six months and had been on antipsychotics for more than eight weeks. The most prescribed medication was quetiapine (n=11), followed by olanzapine (n=4), risperidone (n=2), and haloperidol (n=1). These medications were prescribed for issues like sleep disturbance, anxiety, and agitation, not for mood stabilization in bipolar disorder or augmentation in depression or OCD. Patients were gradually tapered off antipsychotic medications, with quetiapine doses typically reduced by 50% over two months and halved again over the next two months. Olanzapine and risperidone were discontinued over 3-6 months. Those successfully deprescribed reported improved energy, reduced fatigue, and no long-term adverse effects like tremors or extrapyramidal symptoms.

**Conclusions:**

Antispcyhtic deprescribing is an important part of clinical care that reduce the risk to patients and gradual tapering of low-dose off-label antipsychotic medications, including quetiapine, olanzapine, and risperidone, was largely successful in this study, with most patients experiencing improved energy and reduced sedation.

**Disclosure of Interest:**

None Declared

